# AID and APOBECs span the gap between innate and adaptive immunity

**DOI:** 10.3389/fmicb.2014.00534

**Published:** 2014-10-13

**Authors:** Arnaud Moris, Shannon Murray, Sylvain Cardinaud

**Affiliations:** ^1^Center for Immunology and Microbial Infections, Faculty of Medicine, Université Paris-SorbonneUPMC Univ Paris 06, Paris, France; ^2^Center for Immunology and Microbial Infections, Institut National de la Santé et de la Recherche MédicaleU1135, Paris, France; ^3^Center for Immunology and Microbial Infections, Centre National de la Recherche ScientifiqueERL 8255, Paris, France; ^4^Department of Immunology, Hôpital Pitié-SalpêtièreParis, France

**Keywords:** restriction factors, CTL, HIV, correlate of protection, APOBEC1, APOBEC2, APOBEC3

## Abstract

The activation-induced deaminase (AID)/APOBEC cytidine deaminases participate in a diversity of biological processes from the regulation of protein expression to embryonic development and host defenses. In its classical role, AID mutates germline-encoded sequences of B cell receptors, a key aspect of adaptive immunity, and APOBEC1, mutates apoprotein B pre-mRNA, yielding two isoforms important for cellular function and plasma lipid metabolism. Investigations over the last ten years have uncovered a role of the APOBEC superfamily in intrinsic immunity against viruses and innate immunity against viral infection by deamination and mutation of viral genomes. Further, discovery in the area of human immunodeficiency virus (HIV) infection revealed that the HIV viral infectivity factor protein interacts with APOBEC3G, targeting it for proteosomal degradation, overriding its antiviral function. More recently, our and others’ work have uncovered that the AID and APOBEC cytidine deaminase family members have an even more direct link between activity against viral infection and induction and shaping of adaptive immunity than previously thought, including that of antigen processing for cytotoxic T lymphocyte activity and natural killer cell activation. Newly ascribed functions of these cytodine deaminases will be discussed, including their newly identified roles in adaptive immunity, epigenetic regulation, and cell differentiation. Herein this review we discuss AID and APOBEC cytodine deaminases as a link between innate and adaptive immunity uncovered by recent studies.

## INTRODUCTION

Higher eukaryotes have developed multiple strategies to counteract viral infections. A first line of defense is based on the recognition of pathogen-associated molecular patterns (PAMPs) such as viral replication intermediates that are molecules not commonly found in uninfected host cells. PAMPs were originally defined as molecular patterns specific to microbes, highly conserved and required for microbial function, and thus, are self–nonself discriminating molecules for higher eukaryotic organisms. After the engagement of PAMPs with the subsequently identified PAMP receptors, activation of a cascade of events leads to the expression and, in some cases, secretion of antiviral molecules and chemokines. Some of these molecules have been defined as “restriction factors” meaning host factors that have been evolutionarily selected for based on their capacity to restrict microbial infections. The receptors and effectors of this innate immunity are germline-encoded and mediate key aspects of host defense. However, viruses can also evade host defenses. It is the second arm of the immune system, adaptive immunity, which provides flexible antigen recognition based on somatic modification of antigen receptor genes in immune cells. This process involves selection of immune cells that includes a step of deletion of antigen receptors that are self-reactive, thus preventing autoimmunity, while allowing adaptation to diverse pathogens and the establishment of rapid and robust memory responses. There is evidence that communication between innate and adaptive immunity is required to clear pathogen infections that are otherwise deleterious to the host (Iwasaki and Pillai, 2014). Innate and adaptive immunity are thus considered interdependent.

Activation-induced deaminase (AID) and APOBEC (apolipo protein B mRNA editing enzyme, catalytic polypeptide-like) enzymes are important in both innate and adaptive immune responses. AID/APOBEC family members originate from a large gene superfamily encoding for zinc-dependent deaminases involved in the metabolism of purine and pyrimidine bases (Conticello, 2008). The appearance of AID/APOBECs is thought to be concurrent with the divergence of the vertebrate lineage and the evolution of adaptive immunity (Conticello, 2008). AID/APOBECs have a unique capacity to mutate DNA and/or RNA of both host and pathogen as a result of their ability to deaminate cytidine to uridine. This activity, referred to as nucleic acid “editing,” is involved in various immune functions, including restriction of viral replication, antigen presentation, and maturation of host immune receptors. The structure of AID/APOBEC proteins in relation with their editing activities has been recently reviewed (Conticello et al., 2007; Desimmie et al., 2014). AID is thought to be the oldest member of the family and is essential for antigen-driven B cell terminal differentiation and antibody (Ab) affinity maturation and diversification (Muramatsu et al., 2000). In humans, genetic deficiency of AID leads to Type-2 Hyper-IgM Syndrome (HIGM2), an immunodeficiency characterized by the absence of antibodies other than the IgM class (Revy et al., 2000). APOBEC1, the first family member to be identified, plays an important role in lipid metabolism due to its ability to edit the apopoliprotein B (ApoB) pre-mRNA (Navaratnam et al., 1993; Teng et al., 1993). APOBEC1 might also participate in the restriction of viral infections (Ikeda et al., 2008; Gonzalez et al., 2009). APOBEC3s include seven members (A–C, DE, and F–H) that are involved in the restriction of viral infection and propagation affecting viruses such as human immunodeficiency virus (HIV), hepatitis C virus (HCV), and hepatitis B virus (HBV). APOBEC2 and APOBEC4 functions remain poorly understood although a role of APOBEC2 in embryogenesis has been recently proposed (Vonica et al., 2010). Highlighting the crucial role of AID/APOBECs in host defense, viruses have developed mechanisms to interfere with AID/APOBEC biogenesis and/or functions, and in fact, APOBEC3G was originally discovered due to its interaction with the HIV Vif protein (Sheehy et al., 2002). Here, we will review the cellular functions of AID/APOBEC family members and discuss recent work investigating their contribution in innate and adaptive antiviral immunity.

## CELLULAR FUNCTIONS OF AID AND APOBEC FAMILY MEMBERS

### APOBEC1

In humans, APOBEC1 (A1) is uniquely expressed in the gastrointestinal tract and participates in plasma lipid metabolism. In other species, such as mice, rats, horses, and dogs, A1 is also present in the liver (Greeve et al., 1993). Until recently, ApoB pre-mRNA was thought to be the single cellular target of A1 (Teng et al., 1993). ApoB protein has two isoforms, ApoB-100 and ApoB-48, encoded by a single gene in the liver and small intestine, respectively. The shortest form, ApoB-48, is the product of A1 editing activity and corresponds to the N-terminal portion of ApoB. A1 converts a unique cytidine to uridine (at position 6666 in Apo pre-mRNA) leading to a glutamine to STOP codon substitution and ApoB-48 translation (Navaratnam et al., 1993). ApoB-100 and ApoB-48 have different biological properties and control the homeostasis of plasma cholesterol. The editing activity of A1 is therefore an important determinant for plasma concentrations of ApoB-containing lipoproteins that are implicated in development of hyperlipidemia and atherosclerosis. Overexpression of A1 in the liver of mice or rabbits reduces the concentration of low-density lipoproteins. However, A1 overexpression also induces hepatocellular carcinoma in transgenic animals (Yamanaka et al., 1995), most likely due to its capacity to edit DNA (Harris et al., 2002; Petersen-Mahrt and Neuberger, 2003). A1 is indeed expressed in the nucleus where ApoB pre-mRNA editing also occurs (Lau et al., 1991).

More recently, using a transcriptome-wide RNA sequencing screen comparing wild type and A1-deficient mice, Papavasilou et al. discovered that, in small intestine, many mRNA transcripts other than apoB are edited by A1 (Rosenberg et al., 2011). The targets of A1 are 3′-untranslated regions (3′ UTR) of mRNA transcripts, suggesting additional roles for APOBEC1 beyond its function in ApoB regulation.

### APOBEC2

A2 was cloned based on its sequence homology with A1 (Liao et al., 1999). A2 is well-conserved in the vertebrate lineages and can be traced back to bony fish (Liao et al., 1999; Etard et al., 2010). Using *in vitro* models (e.g., *Escherichia coli*), A2 has been shown to exhibit intrinsic cytidine deaminase activity (Liao et al., 1999). Although the A2 structure has been solved (Prochnow et al., 2007), its functions remained elusive until recently. In humans, A2 is exclusively expressed in heart and skeletal muscles (Liao et al., 1999). In mice, A2 KO was reported to have no major effect on animal viability and fertility (Mikl et al., 2005). This is in contrast to recent studies that implicate A2 in embryonic development of fish and xenopus (Etard et al., 2010; Pennings et al., 2010; Vonica et al., 2010). The lack of A2 expression causes a dystrophic muscle phenotype in zebrafish embryos (Etard et al., 2010). A2 seems to inhibit TGFβ-signaling, thus promoting muscle fiber differentiation both *in vivo* (in zebrafish and xenopus embryos) but also *in vitro* using a mammalian myoblastic cell line (Vonica et al., 2010). The mechanism of action and the targets of A2 action during embryogenesis are not defined, however, the ability of A2 (and other deaminases such as AID) to deaminate methylated cystidines suggests a possible role in epigenetic regulation (Rai et al., 2008).

### AID

Activation-induced deaminase was cloned in a subtractive cDNA library screen comparing activated and resting B cell lymphomas (Muramatsu et al., 1999). AID is a key determinant in the generation of protective Ab-mediated adaptive immune responses. The cytidine deaminase activity of AID initiates the introduction of double stranded DNA breaks (DSB) in the immunoglobulin heavy chain (IgH) gene locus allowing Ab diversification, referred to as class switch recombination (CSR; Muramatsu et al., 2000). In addition, AID produces point mutations at the V(D)J region of Ig loci, a mechanism referred to as somatic hypermutation, (SHM), allowing B cell maturation (Muramatsu et al., 2000). These functions require a rigorous targeting of AID activities to SHM and CSR substrates (Kohli et al., 2010). Targeting might involve several complementary mechanisms such as AID binding to replication protein A, a ssDNA-binding protein involved in DNA repair (Basu et al., 2005), and/or association with a non-encoding RNA-processing/degradation complex (Basu et al., 2011). The editing activity of AID is not restricted to Ig loci and AID can act on a wide spectrum of genomic targets in B cells (Yamane et al., 2011). As a consequence, aberrant expression of AID promotes cancer development in animal models and humans (Okazaki et al., 2007). Dysregulated expression of AID facilitates DNA translocations that require DSB such as *c-myc/IgH* found in Burkitt’s lymphoma and *c-myc/miR-142* found in B cell leukemia (Robbiani et al., 2008, 2009; Hasham et al., 2010). Constitutive or ubiquitous AID expression also leads to cancer development that is characterized by point mutations in oncogenes as well as passenger mutations (those mutations that do not contribute to cancer growth; Okazaki et al., 2003). AID can therefore produce mutations in many genes other than Ig genes. While most of these mutations are rapidly repaired by the cellular DNA-repair machinery, those that are not successfully repaired, can destabilize the genome of cells.

Although AID expression is at its highest levels in germinal center B cells that undergo CSR and SHM, it is also found in other cell types such as oocytes, embryonic stem (ES) cells, and in estrogen-induced breast tissue (Fritz and Papavasiliou, 2010). The function of AID expression in these cells or tissues remains to be elucidated. However, the study of lower vertebrates including zebrafish suggests that AID expression is involved in epigenetic reprogramming of germ cells during early development (Rai et al., 2008). Using an AID knockout mouse model, Popp et al. (2010) revealed a role of AID in DNA demethylation during primordial germ cell reprogramming. DNA cytosine methylation is associated with gene silencing and plays a key role in development and genomic imprinting. The removal of 5-methyl group on cytosine (5-mC) contributes to epigenetic reprogramming required for the restoration of pluripotency of germ cells. Several lines of evidence suggest that AID, but also A1 and A2, might participate in this process of demethylation: AID and A1 can deaminate 5-methylcytosine *in vitro* and in *E. coli* (Morgan et al., 2004), and germ cells from AID-deficient mouse exhibit a hypermethylation pattern (Popp et al., 2010). AID (and A2) might contribute to the conversion of 5-mC to thymidine (T) later replaced by cytosine (C) by the DNA-repair machinery (Rai et al., 2008). In summary, AID function is not limited to Ab diversification, and evidence is accumulating to suggest a role in epigenetic reprogramming.

### APOBEC3s

Sheehy et al. (2002) initially discovered the first family member of APOBEC3, A3G, in ground-breaking studies with HIV infection. Since that original identification, seven human A3 genes clustered in tandem on chromosome 22 have been identified, namely, A3A, A3B, A3C, A3DE, A3F, A3G, and A3H, which most likely arose through gene duplication of a single-copy primordial gene (Jarmuz et al., 2002). A3E was thought to be a pseudogene but in fact, A3D and A3E form one unique protein (A3DE; Dang et al., 2006). All A3 genes encode one or two conserved zinc-coordinating deaminase domain (ZDD), which contains a His/Cys-Xaa-Glu-Xaa_23-28_-Pro-Cys-Xaa_2-4_-Cys signature motif [X denotes any amino acid (aa)]. Regions of human A3 mRNAs share between 30 and 100% homology. Interestingly, depending on the species, the A3 genes expanded and/or contracted. As a result, A3 gene number ranges from one (mice, rats, pigs) to three (cats) and six (horses; LaRue et al., 2008). In humans, A3 genes are also highly polymorphic most likely due to the fact that they have been under strong and continuing selective pressure during primate evolution (Conticello et al., 2005; Henry et al., 2012). As discussed later on in this review, A3 polymorphisms might influence their specific antiviral activity.

A3 gene expression has been mainly documented in immune cells and these results have been determined based on mRNA levels in cells, using quantitative PCR (Koning et al., 2009; Refsland et al., 2010). This approach is particularly difficult since A3 genes are highly homologous and polymorphic. Apart from A3G and A3A, most antibodies to A3s are not very specific and endogenous A3 proteins are often difficult to detect. Nonetheless, several studies indicate a strong correlation between mRNA level and protein expression (Refsland et al., 2010). There is a general consensus that most A3s are highly expressed in T cells [memory or naïve (Refsland et al., 2010)] but also in B cells and phagocytic cells. A3A and A3B are predominantly expressed in monocytes (Peng et al., 2007; Thielen et al., 2010) and B lymphocytes (Koning et al., 2009), respectively. A3G and A3F are expressed in T cells, monocytes and dendritic cells (DC; Sheehy et al., 2002; Pion et al., 2006; Peng et al., 2007; Pido-Lopez et al., 2007; Stopak et al., 2007; Trapp et al., 2009). However, there is no consensus regarding their relative abundance. A3s expression is not confined to immune cell populations, and are highly expressed in human testis and ovary (A3G and A3F) (Koning et al., 2009) as well as ES cells (A3B, A3C, A3DE, A3F, and A3G; Wissing et al., 2011). A3G, A3F, A3B, and A3C are expressed in primary hepatocytes (Bonvin et al., 2006; Tanaka et al., 2006). The true breadth of basal A3 expression in human tissues remains difficult to estimate as leukocytes infiltrate tissues and no suitable specific immunohistochemistry antibodies are presently available. Nevertheless, different observations are in favor of a broad and constitutive A3 expression profile in human tissues. For instance, various cancer cell lines of non-immune origin - colorectal adenocarcinoma, melanoma and lung carcinoma lines - express multiple human A3s. It is possible that A3 expression is induced during oncogenesis, but given the abundance of A3s in different cell types it might also reflect their normal expression profile prior to cell transformation.

The cellular expression of A3s clearly indicates a role of A3s in immunity. The broad distribution of A3s also points toward a putative role in cellular maintenance. A3G and A3F localize in cytoplamic microdomains and stress granules that are sites of RNA storage and metabolism also called mRNA-processing bodies (or P-bodies; Wichroski et al., 2006; Gallois-Montbrun et al., 2008). Within P-bodies, A3G and A3F interact with effectors of the RNA silencing machinery (such as Argonaute 1 and 2) and translation suppressor (RCK/p54), suggesting that A3G-F participate in RNA metabolism and fate determination (Wichroski et al., 2006; Gallois-Montbrun et al., 2008). However, Phalora et al. (2012) found no evidence that A3s participate in specific regulation of miRNA. In addition, the manipulation of P-bodies using siRNA inhibition had no impact of A3 antiviral functions and HIV replication (Phalora et al., 2012). The reason why A3G and A3F localize to these P-bodies remains unclear. More recently, a role of A3s in DNA catabolism has also been proposed. Reminiscent of AID capacity to deaminate B cell genomes (during SHM and CSR), A3A edits host nuclear and mitochondrial DNA leading to the introduction of uridine (Suspene et al., 2011a). In the presence of functional DNA repair machinery, most mutations are likely fixed. In contrast, in uracil DNA-glycosylase (UNG)-deficient cells, (UNGs are enzymes required for excision of uracil bases), cytidine deaminations are readily detected using differential DNA-denaturation PCR (3D-PCR) (Suspene et al., 2011a). The significance of nuclear DNA editing by A3A is rather enigmatic as hyperediting is synonymous with cell death and aberrant editing and/or repair might contribute to tumorigenesis (Mussil et al., 2013). On the other hand, phagocytic cells that express predominantly A3A may use cytidine-deamination to mark foreign DNA for degradation. In this model, the deamination of multiple cytidines on foreign DNA might lead to uracil excision by UNG, creating nuclease-sensitive abasic sites, and subsequent degradation by cellular nucleases (Stenglein et al., 2010). The nucleases involved have not been characterized, but as discussed by Stenglein et al. (2010) might include the IFN-inducible APEX or TREX1, though a contribution of DNAse I and II cannot be ruled out. This mechanism might represent an intrinsic immune defense reminiscent of bacteria that evolved endonucleases to prevent DNA transmission and bacteriophage infection (Stenglein et al., 2010). To this regard it is interesting to note that A3A and other A3s are induced upon inflammation (as described further, below).

Much remains to be learned regarding the cellular functions of A3s. Depending on cell type and tissue environment, A3s differently contribute to DNA/RNA deamination and their overarching biological roles are still being elucidated.

## AID, APOBEC1, AND APOBEC2 IN ANTIVIRAL IMMUNITY

### APOBEC1

The sequence homology between A1 and A3G prompted researchers to investigate a potential role of A1 in viral infection (Bishop et al., 2004a,b). In a pioneering work, Bishop et al. (2004b) demonstrated that human A1 (hA1) incorporated into HIV particles had no effect on HIV replication. In contrast, rat A1 had a strong suppressive effect on HIV regardless of Vif expression (Bishop et al., 2004b). Later work confirmed that in contrast to hA1, A1 from small animals (e.g., rabbit, hamster, mouse) inhibited the replication of retroviruses such as SIV (simian immunodeficiency virus), FIV (feline immunodeficiency virus), and murine leukemia virus (MLV), and the activation of autonomous retroelements in a deaminase-dependent manner, thus suggesting a putative role for A1 in the restriction of viral replication (Ikeda et al., 2008). The demonstration that A1 is a restriction factor in the course of viral infections in natural hosts came from the study of MLV and hepadnaviruses by the group of Wain-Hobson and Vartanian (Petit et al., 2009; Renard et al., 2010). Analyzing viral sequences in HBV-infected chimpanzees, woodchucks chronically infected with the natural woodchuck hepatitis virus (WHV) as well as ducks infected with duck hepatitis virus (DHV), the authors provided evidence that A1 edits hepadnaviral genomes and restricts replication *in vivo* (Renard et al., 2010). Analyzing human serum from two HBV chronically infected carriers, the same group also suggested that A1 edits HBV genomes *in vivo* (Gonzalez et al., 2009). These results were somehow surprising due to the fact that in humans A1 is not normally expressed in the liver. However, viral infection might lead to ectopic expression of A1. During the course of viral infections, the influence of IFN induction (or treatment) on A1 expression has not been investigated thus far. Nonetheless, the function of A1 is most likely not limited to the regulation of lipid metabolism. In vertebrates, A1 likely participates in intrinsic defenses against some viral infections.

### APOBEC2

Though A2 exhibits deaminase activities (Liao et al., 1999), it has not been assigned a role in the restriction of viral replication thus far. However, it is interesting to note that in hepatocytes, A2 expression is enhanced by pro-inflammatory cytokines such as TNFα and IL-1β (Matsumoto et al., 2006). A2 contains functional NF-kB response elements in the 5′ untranslated region, suggesting a possible involvement in immune responses (Matsumoto et al., 2006). In the tonsils of patients with Immunoglobulin A nephropathy (IgAN) a disease characterized by IgA deposition to glomerular mesangial cells and glomerulonephritis, A2 expression is up-regulated around B cell germinal centers (where B cells undergo CSR and SHM with the “help” of follicular T cells). However, a direct role of A2 in IgAN pathology or IgA production has not been established (Iio et al., 2010).

### AID

As discussed earlier, AID is required for CSR and, as a result, is critical for the generation of B cells that secrete Abs with various effector functions and tissue distribution in the organism (Muramatsu et al., 2000). For instance, immunoglobulins of the IgA isotype are found at the portal of pathogen entry in the mucosa and can be transported across the epithelium to neutralize pathogens. IgG is the principal isotype in the blood and extracellular fluid and is involved in pathogen neutralization, opsonization, and complement activation. *AID* ^-/-^ mice harbor a complete defect of CSR with a hyper-IgM phenotype and present enlarged germinal centers containing activated B cells (Muramatsu et al., 2000). In addition, AID involvement in SHM allows the generation of B cells with the potential to secrete Abs with higher affinities (Imai et al., 2003). Interestingly, mice carrying a mutated allele of AID with reduced capacity to perform SHM but with normal amounts of CSR, exhibit an impaired gut homeostasis and inefficient mucosal defenses (Wei et al., 2011). In humans, genetic deficiencies of AID are responsible for the development of a rare immunodeficiency, HIGM2 (Revy et al., 2000). HIGM2 is characterized by the absence of antibodies other than IgM and a profound susceptibility to bacterial infections (Revy et al., 2000). AID is therefore a key determinant in protective immunological responses, and the most well-documented mechanism of this protection is through the generation of protective Ab-mediated immune responses.

The action of AID is not limited to B cell differentiation and maturation as there is accumulating evidence that AID contributes to innate defenses against viruses. For example, HCV, Epstein-Barr virus (EBV), and Kaposi’s sarcoma-associated herpesvirus (KSHV) have been shown to induce AID expression in B cells residing outside the germinal centers (Machida et al., 2004; Rosenberg and Papavasiliou, 2007; Bekerman et al., 2013). It is unclear so far whether AID up-regulation is beneficial or deleterious to HCV and EBV, however, in the case of KSHV, AID has a direct impact on viral fitness by inhibiting lytic reactivation and by reducing infectivity of virions. Further reinforcing the role of AID in antiviral responses, KSHV encodes microRNAs that dampen AID expression (Bekerman et al., 2013). Whether the deaminase activity of AID is required for KSHV restriction [as described for A3G (see below)] remains to be determined. In hepatocytes, AID expression also correlates with reduced susceptibility to HBV infection (Watashi et al., 2013), a mechanism that might be dependent on deamination of the HBV genome by AID (Liang et al., 2013). AID might also participate in responses against transforming retroviruses. AID-deficient mice have been shown to be more susceptible to Abelson murine leukemia virus (A-MuLV), a defective virus that causes pro-B cell leukemia *in vivo* (Gourzi et al., 2006). In this case, the action of AID does not involve direct editing of the viral genome. Instead, AID might cause damage in the host cell genome, resulting in cell cycle arrest and/or up-regulation of stress-inducible factors, leading to natural killer (NK) cell activation and thus slower tumor growth (Gourzi et al., 2006). AID expression also correlates with the induction of aberrant SHM that might contribute to B cell transformation and tumorigenesis (Machida et al., 2005; Epeldegui et al., 2007).

## APOBEC3s AND ANTIVIRAL IMMUNITY

A3G was the first member of APOBEC family to be assigned a role in antiviral immunity by demonstration of its activity against HIV infectivity (Sheehy et al., 2002). Since then, it has been demonstrated that human A3 cytidine deaminases affect the replication of a variety of viruses (Chiu and Greene, 2008) and impact the activation of adaptive immunity (Casartelli et al., 2010). A3G restricts the replication of retroviruses such as HIV, Foamy virus (FV; Delebecque et al., 2006), human T-cell leukemia virus type-1 (HTLV-1; Mahieux et al., 2005) as well as DNA viruses such as HBV (Turelli et al., 2004; Suspene et al., 2005) and also affects endogenous retroviruses (Esnault et al., 2005). A3A, A3C, and A3H deaminate human papillomavirus (HPV) genomes (Vartanian et al., 2008) and A3C acts on herpes viruses [e.g., herpes simplex-1 (HSV-1) and EBV viruses (Suspene et al., 2011b)]. Human A3G also acts on viruses infecting rodents (MLV) or avian species (Rous sarcoma virus and alpharetroviruses). A3 family members can play redundant roles in antiviral immunity (Albin and Harris, 2010). For instance, A3G and A3F restrict HIV and like A3C, A3G also acts on herpes viruses, although to a lesser extent (Suspene et al., 2011b). Intrinsic specificities of A3 proteins, but also their tissue distribution and cellular expression levels most likely determine the impact of each A3 family member on viral replication and on the activation of antiviral immunity.

## INTRINSIC ANTIVIRAL FUNCTION OF A3s

**FIGURE 1:**
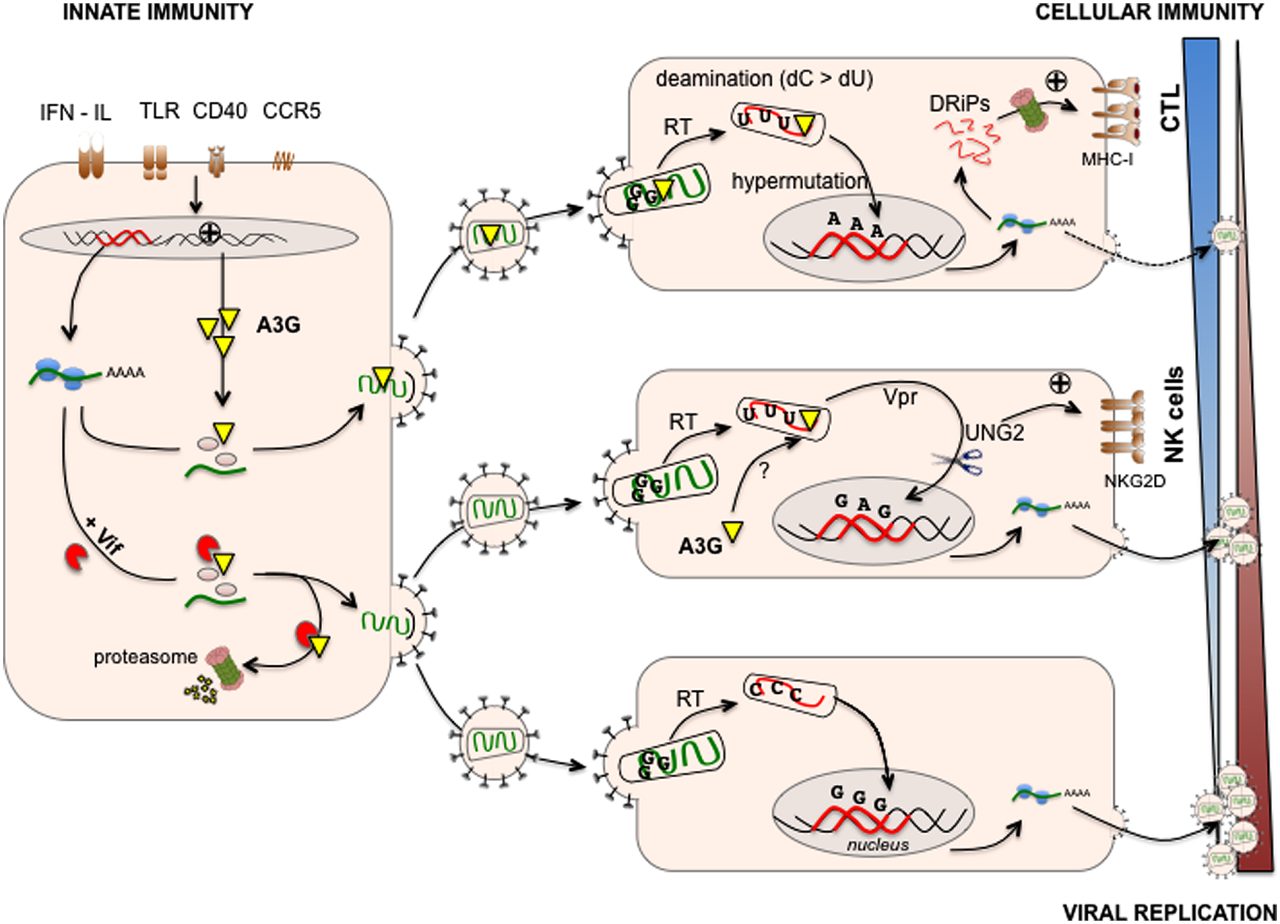
**APOBEC3G links innate/intrinsic immunity with adaptive cellular immunity.** A3G is expressed at various levels in human immunodeficiency virus (HIV) infected cells and can be induced upon viral sensing by Toll-like receptors (TLR), triggering of CD40 or CCR5 and IFN production **(left)**. A3G is incorporated in budding virions and exerts its antiviral activities in newly infected cells **(right)**. During reverse transcription, A3G catalyzes cytosine to uracil deaminations on the nascent minus DNA strand of HIV. The presence of uracil and the expression of HIV Vpr recruit the cellular DNA repair machinery (UNG2, **right middle**) leading to either HIV DNA restoration or degradation, the latter reducing viral replication. The cellular DNA-repair machinery trying to cope with uracil-rich HIV DNA also activates the DNA-damage- and stress–response pathways leading to the up-regulation of activating natural killer (NK) cell ligands (NKG2D ligands) and killing of infected cells **(right middle)**. In addition, deaminations lead to G to A transitions in HIV DNA leading to the integration of proviruses with a high frequency of amino acid substitutions and/or premature STOP codons. These proviruses express aberrant viral proteins that are unable to produce infectious particles. Infected cells can use these misfolded or truncated viral proteins (called DRiPs, defective ribosomal products) to generate MHC-I epitopes leading to activation of anti-HIV cytotoxic T lymphocyte (CTL) responses **(right upper)**. However, in infected cells, Vif counteracts the antiviral functions of A3G by targeting newly synthetized A3G for proteasomal degradation, thus reducing the amount of A3G incorporated into budding virions **(left)** and facilitating HIV replication in newly infected cells **(right bottom)**. Viral replication is therefore the result of a balance between anti-viral innate and adaptive (cellular) immunity promoted by A3G and Vif-mediated escape mechanisms developed by HIV.

The characterization of mutant HIV defective for the accessory protein Vif led to the discovery of A3G. Vif is essential for HIV-1 replication in a variety of cells including primary human T cells, monocytes, macrophages, DC and lymphoid T cell lines such as CEM, HUT78, also called “non-permissive” cells. However, Vif is dispensable in “permissive” T cell lines such as CEM-SS (a variant of CEM), Jurkat and supT1 cells [see for an in depth review (Henriet et al., 2009)]. A subtractive cDNA library screen – using CEM and CEM-SS T cell lines - led to the identification of human A3G that is strongly expressed by non-permissive CEM-SS cells (Sheehy et al., 2002). In non-permissive cells, A3G is incorporated into budding virions and acts on HIV replication in a post-fusion event in newly infected cells (**Figure 1**). Transfection of A3G in permissive cells leads to abrogated replication of Vif-deficient HIV (HIV Δ*Vif)* (Sheehy et al., 2002) and sequencing of HIV Δ*Vif* DNA revealed a hypermutated pattern with enriched G to A transitions (Lecossier et al., 2003), strongly suggesting that A3G deaminase activity is required for the restriction of HIV replication (Zhang et al., 2003). It is now established that Vif counteracts the antiviral functions of A3G and other A3 family members such as A3F and a certain allele of A3H (Henriet et al., 2009). The action of Vif on A3s has been extensively reviewed (Henriet et al., 2009), but in sum, in infected cells, Vif targets A3G for proteasomal degradation, reducing the amount of A3G incorporated into budding virions (**Figure 1**). Indeed, as a post-fusion event, A3G catalyzes cytosine to uracil deamination on the nascent minus DNA strand of the HIV reverse transcribed genome (Harris et al., 2003; Mangeat et al., 2003; Yu et al., 2004). The presence of uracil on the minus strand of HIV DNA might target HIV DNA for degradation by the cellular DNA repair machinery thus reducing viral replication (Mariani et al., 2003). As exemplified by the existence of hyper-edited sequences retrieved from HIV proviruses *in vivo* (Kieffer et al., 2005), the action of Vif on A3G incorporation/degradation is not absolute and deaminations also lead to G to A transitions in HIV DNA (Harris et al., 2003; Lecossier et al., 2003; Mangeat et al., 2003; Yu et al., 2004). Editing patterns are dominated by GG to AG hypermutations leading to a high frequency of amino acid substitutions and to the introduction of premature STOP codons (Vartanian et al., 1991). These crippled proviruses express aberrant (i.e., misfolded or truncated) viral proteins that are unable to produce infectious particles (Simm et al., 1995).

Shortly after the discovery of A3G, A3F was shown to restrict HIV replication. A3G and A3F editing is not random. A3G and A3F preferentially introduce mutations in TGG or TGA sequences, respectively. There is a lack of consensus on the ability of other A3 family members to edit HIV [see for a review (Albin and Harris, 2010)]. For instance, over-expression of A3B and A3DE exerts anti-HIV activity in a single cycle assay but insignificant antiviral activity in a spreading infection system (Hache et al., 2008). A3D, F, G, and H but not A3B, C, and DE restrict infection of human CD4+ T lymphocytes by Vif-deficient viruses (Hultquist et al., 2011). A3DE restricts HIV replication in macrophages but to a lower extent than A3G (Chaipan et al., 2013). In myeloid cells, APOBEC3A blocks early steps of reverse transcription but acts when expressed in the target cell of infection (Berger et al., 2011).

There is a general agreement that cytidine deamination plays an important role in the capacity of A3s to restrict viral replication (Schumacher et al., 2008; Browne et al., 2009). However, several reports also suggest that A3G and A3F restrict viral replication to a significant extent through deaminase-independent mechanisms (Newman et al., 2005). A3G and A3F affect reverse transcription priming and extension (Mangeat et al., 2003; Mariani et al., 2003; Guo et al., 2006; Anderson and Hope, 2008; Malim, 2009; Wang et al., 2012; Gillick et al., 2013) and HIV DNA integration into the host genome (Luo et al., 2007; Mbisa et al., 2007; Vetter and D’Aquila, 2009). As proposed by Henriet et al. (2009) interference of A3G and A3F with the viral core assembly might be responsible for these deaminase-independent impairments of HIV replication. The balance of editing and non-editing-dependent effects of A3G and A3F varies depending on the experimental system and might be affected by their cellular expression levels (Miyagi et al., 2007; Knoepfel et al., 2008; Browne et al., 2009).

## INDUCTION OF A3 EXPRESSION

In humans, A3s are not only expressed in a variety of tissues, but their expression is also induced by mediators of inflammation, possibly reflecting their role as a first line of defense against invading viruses (**Figure 1**). IFN-α was reported to enhance A3G and A3A expression in monocytes and macrophages. IFN-γ and -β also induce A3G up-regulation in macrophages (Sarkis et al., 2006; Peng et al., 2007; Stopak et al., 2007; Koning et al., 2009; Refsland et al., 2010; Berger et al., 2011). IFN-α secreted by plasmacytoid dendritic cells (pDC) enhances the expression of A3A, A3C, A3G, and A3F within pDC, indicating that pDC might be armed against viral infection by an autocrine IFN-α loop (Wang et al., 2008). Pathogen sensors such as Toll-like receptors (TLR) also influence A3 gene expression. TLR-3 stimulation by the double stranded RNA analog [poly(I:C)] induces type I IFN responses in DC and subsequent A3G expression (Trapp et al., 2009). Overall, the induction of DC maturation using stimuli such as LPS (a TLR-4 ligand), CCR5 and CD40 ligands correlates with the up-regulation of A3G (Pion et al., 2006; Pido-Lopez et al., 2007; Stopak et al., 2007). The effect of IFN-α on A3 expression in primary CD4+ T cells is controversial, but in most reports no induction was observed (Rose et al., 2005; Chen et al., 2006; Sarkis et al., 2006; Stopak et al., 2007; Ying et al., 2007; Koning et al., 2009; Refsland et al., 2010). In contrast, IL-2, IL-7, IL-15, and IL-27 and mitogens such as phytohemagglutinin (PHA) and phorbol myristate acetate (PMA) induced modest and strong activation of A3G expression, respectively, (Stopak et al., 2007). Combined with IL-2, PHA induces expression of all A3s except A3A (Greenwell-Wild et al., 2009; Koning et al., 2009; Refsland et al., 2010). Triggering of the T cell receptor (TcR) also induces A3G expression in effector memory T cells and interferes with HIV replication *in vitro* (Pido-Lopez et al., 2009).

The induction of A3 expression is not limited to immune cells. IFN-α secretion, for instance, can induce A3G, A3F, and A3B expression in hepatocytes (Bonvin et al., 2006; Sarkis et al., 2006; Tanaka et al., 2006). In contrast, other factors reduce A3 protein expression. The nerve growth factor (NGF), an essential factor for survival and activation of monocytes/macrophages, is released by HIV infected macrophages and dampens the synthesis of A3G, overriding the IFN-γ-induced upregulation of A3G (Souza et al., 2011).

Overall, the transcriptional regulation of A3 genes seems to play a major role in A3-dependent defenses against viruses. Remarkably, following mucosal immunization of nonhuman primates, two studies reported the up-regulation of A3G in CCR5+ CD4+ memory T cells (Wang et al., 2009) as well as in mucosal DC and CD14+ cells (Sui et al., 2010), all potential cellular targets of HIV replication. A3G mRNA upregulation was maintained over several weeks after immunization and upon challenge with SIV, and correlated inversely with viral loads and positively with a better preservation of CD4+ T cells in the gut (Sui et al., 2010). These studies strongly suggest that A3G provides an antiviral effect *in vivo*. They also demonstrate that mucosal immunization triggers an innate signature that promotes adaptive immune responses. The exact mechanism underlying vaccine-induced A3G expresssion with protection from SIV infection is not clear. As observed in the murine model of Friend retrovirus (FV) infection (Santiago et al., 2010), by limiting early viral replication, A3G might delay virus-induced immune dysfunction and thus might favor the establishment of humoral [e.g., generation of FV-neutralizing antibodies (Santiago et al., 2010)] and cellular immunity.

## A ROLE OF A3G IN THE ACTIVATION OF CELLULAR AND ADAPTIVE IMMUNITY

There is growing evidence that A3G bridges intrinsic and cellular immunity as is the case with AID, that shapes the Ab repertoire, blocks virus-induced cancer by inducing cell cycle arrest and activates NK cells (Gourzi et al., 2006). A3G has been implicated in enhancing NK-cell killing of HIV-infected T cells (Norman et al., 2011). This process requires A3G-editing of HIV DNA (Norman et al., 2011). In this study, the authors propose that the cellular DNA-repair machinery activates DNA-damage- and stress–response pathways in response to uracil-edited HIV DNA, leading to the up-regulation of activating NK cell ligands (NKG2D ligands) and killing of HIV infected cells. The authors also implicate the HIV Vpr protein and its capacity to recruit the DNA-repair machinery as a key factor in NKG2D ligand expression. In contrast, HIV Vif counteracts the action of A3G and the NK cell-mediated elimination of HIV-infected cells (Norman et al., 2011). Interestingly, in the work of Norman et al. (2011), cytoplasmic A3G (expressed by the target cell) seems responsible for HIV editing and thus NK cell recognition. Cytoplasmic A3G has been shown to impact HIV genome integration but has not been assigned a role in HIV genome editing, to our knowledge (Vetter and D’Aquila, 2009). Whether the accumulation of unintegrated HIV genomes or the editing of HIV genomes *per se* might be responsible for the induction of stress responses and thus NK cell recognition remains to be clarified (Norman et al., 2011). Nonetheless, this study suggests that A3G antiviral functions allow triggering of danger signals that, in turn, activate effectors of cellular immunity.

A3G-mediated editing also contributes to the activation of effectors of adaptive immunity (Casartelli et al., 2010), namely, CD8+ cytotoxic T cells (CTLs). CTLs, whose function is critical in the control of HIV infection, are triggered by viral peptides presented by MHC class-I molecules on the surface of infected cells. These antigenic peptides originate from proteasomal degradation of native viral proteins and of defective/abortive proteins generated in the course of translation (Yewdell and Nicchitta, 2006). We made the assumption that hyper-edited proviruses that express aberrant – misfolded or truncated – viral proteins might represent a source of HIV antigens. We demonstrated that A3G enhances cytotoxic T lymphocyte (CTL) recognition of HIV-infected cells (Casartelli et al., 2010). This process requires A3G-editing of HIV DNA as editing–deficient A3G mutants failed to promote CTL activation. Mimicking A3G-editing, we also showed that truncated HIV proteins represent a major source of HIV antigen for CTL activation (Casartelli et al., 2010). However, and as expected, Vif as well as Nef (that down-regulated MHC-I expression) counteracts the action of A3G in antigen generation. Therefore, recognition of HIV-infected cells by CTLs results from a balance between the efficacy of antigen generation enhanced by A3G editing and the immune-escape mechanisms mediated by the virus. Overall, these studies demonstrate that A3G-mediated viral restriction contributes to the immunogenicity of HIV-infected cells and to NK cell as well as CTL activation, thus linking innate and adaptive immunity (**Figure 1**). Whether other APOBEC3 family members enhance NK cell and CTL activation remains to be determined.

## *IN VIVO* INTERACTIONS OF APOBEC3s AND HIV-1

### A3s AND CORRELATES OF DISEASE PROGRESSION

Accumulating evidence suggests that A3s play an important role in limiting viral replication *in vivo*. APOBEC3 genes have been subject to strong positive selection throughout the history of primate evolution (Sawyer et al., 2004). In persistent HBV infection, A3 polymorphisms seem to impact liver disease progression and viral loads (Ezzikouri et al., 2013). In the course of HIV infection, A3G plays a critical role in limiting the viral reservoir. A major barrier to an effective cure of HIV infection is the maintenance of a latent viral reservoir in the memory CD4 T cell compartment within HIV-infected, successfully treated (ST) individuals (Chomont et al., 2009). Examining the state of the latent proviruses in these cells from ST subjects, several studies have established that approximately 30% of HIV proviruses that can not be reactivated *in vitro* carry the hallmark of A3G editing (Ho et al., 2013). Thus, *in vivo*, A3G plays a significant role in rendering the persistent viral reservoir defective, inactive, or “non-inducible.” However, whether A3G antiviral functions play a critical role in HIV progression is less clear (Albin and Harris, 2010). Analyzing the mutation patterns of proviral sequences isolated from various cohorts, several studies showed a correlation between hypermutation mediated by either A3G or A3F and reduced viral loads (Pace et al., 2006; Vazquez-Perez et al., 2009; Kourteva et al., 2012) or increased CD4 counts (Land et al., 2008). Consistent with these observations, proviral sequences from HIV-infected long-term non-progressors (LTNP) or viral controllers (elite controllers) seem to harbor an elevated level of hypermutation compared to antiretroviral therapy (ART)-treated or naïve non-controllers (Kourteva et al., 2012; Eyzaguirre et al., 2013). In contrast, others observed no correlation between hypermutation with markers of disease progression (Piantadosi et al., 2009; De Maio et al., 2012) nor with the clinical status such as being elite controllers (Gandhi et al., 2008). These contrasting results might be due to technical issues or the size of the cohorts studied.

However, owing to the editing-independent antiviral activities of A3s, other studies analyzed the association between A3G or A3F expression levels (mostly at the mRNA level) and markers of disease progression. But, again, there is no clear-cut answer. Some studies observed that the mRNA levels of A3G correlate positively with CD4 cell counts (Jin et al., 2005; Vazquez-Perez et al., 2009; Zhao et al., 2010) and inversely with viremia (Jin et al., 2005; Zhao et al., 2010; Kourteva et al., 2012) or the viral set point (that is predictive of disease progression; Ulenga et al., 2008). In addition, exposed uninfected individuals showed greater A3G mRNA levels (Biasin et al., 2007; Vazquez-Perez et al., 2009) and controllers with high A3G protein expression in CD4 T cells seem to harbor fewer HIV proviruses (De Pasquale et al., 2013). However, others observed no correlation between mRNA expression levels of either A3G or A3F and markers of disease progression (Cho et al., 2006; Amoedo et al., 2011). Also, during primary infection no association between A3G expression and viral loads was observed (Reddy et al., 2010) and others did not find greater A3G mRNA levels in exposed uninfected individuals (Mous et al., 2012). Larger cohort studies analyzing side-by-side hypermutation patterns and expression levels of all A3 family members might help in establishing a correlation with clinical parameters.

However, variants of A3 at the genetic levels might also account for limiting disease progression. In a pioneering study, An et al (An et al., 2004) identified a variety of A3G polymorphisms within introns and exons that correlate with clinical parameters. A3G H186R identified in this study was shown in various cohort studies to correlate with disease progression (An et al., 2004; Reddy et al., 2010; Singh et al., 2013). The variant H186R of A3G is also associated with low CD4 counts (Bunupuradah et al., 2012) and a polymorphism within an A3G intron (C40693T) was shown to correlate with increased risk of infection (Valcke et al., 2006).

Other members of the APOBEC3 family present common polymorphisms such as A3H and A3B. In contrast to A3G, A3H anti-HIV activity is strongly influenced by its polymorphisms (Ooms et al., 2013) with some specific alleles associated with lower viremia (Gourraud et al., 2011). A3B is deleted in ∼20% of the world’s population. An early study based on a large number of U.S. patients showed that a homozygous deletion of A3B was associated with increased susceptibility to HIV acquisition and progression to AIDS (An et al., 2009). However, a more recent study on Japanese individuals did not observe such a correlation (Imahashi et al., 2014).

Whether A3 family members influence viral transmission and disease progression remains an open question. In addition, the underlying mechanisms are not clear, such as whether A3G H186R and C40693T differentially impact HIV replication, and to date, this has not been experimentally demonstrated.

## A3-MEDIATED EDITING AND VIRAL DIVERSIFICATION/ADAPTATION

A3 antiviral activities, and, in particular, editing, might also facilitate HIV survival by introducing sub-lethal mutations that, in turn, favor HIV diversification and adaptation to ART and/or immune responses. The action of Vif on A3 degradation is not absolute thus allowing A3 incorporation and subsequent sub-lethal editing (Sadler et al., 2010). Evidence of this phenomenon was provided by the demonstration that a Vif allele carrying the K22H mutation, less effective in counteracting A3-mediated editing, was prevalent in a cohort of ART-treated patients experiencing virological failure (Fourati et al., 2010). In this study, several drug resistance mutations in reverse transcriptase (RT) and in the protease, were significantly more common in patients harboring elevated levels of K22H-mutated viruses (Fourati et al., 2010). The expression of K22H-Vif might favor adaptation to antiviral drugs by allowing residual A3-mediated deamination and introduction of mutations into HIV genome. A parallel might be drawn here with hypermutator strains of bacteria that adapt more rapidly to antibiotic. These hypermutator strains of bacteria have mutations in genes affecting DNA repair and replication fidelity and exhibit elevated mutation rates (Woodford and Ellington, 2007). Cytidine deamination might directly promote mutations that generate resistance to drugs. For example, the common M184V mutation of RT that causes resistance to 3TC is located in an A3G editing hotspot and is produced *in vitro* by A3G during HIV replication (Mulder et al., 2008; Kim et al., 2010). A3G-mediated sub-lethal editing might also favor mutations within sequences encoding HIV CTL epitopes thus allowing the virus to escape CTL-recognition (Wood et al., 2009; Monajemi et al., 2014). In contrast, cytidine deamination outside HIV CTL epitopes (especially downstream of the epitope) might favor the expression of unstable aberrant or truncated HIV proteins and thus HIV-specific CTL activation (Casartelli et al., 2010).

On the other hand, A3G-editing at physiological levels might be simply lethal to HIV. Armitage et al. (2012) showed, in an *in vitro* study, that even a single A3G molecule within one HIV particle is likely to cause extensive and inactivating levels of HIV hypermutation (Armitage et al., 2012). The authors suggest that A3-mediated hypermutation might be a discrete “all or nothing” phenomenon (Armitage et al., 2012). The overall impact of A3-mediated hypermutation on HIV survival and adaptation might also vary depending on the anatomical site (Fourati et al., 2014).

## CONCLUDING REMARKS

The AID-APOBEC family members evolved from a family of cytidine deaminases to fight viral infection. They constitute an important arm of innate immunity by restricting replication and spread of a plethora of viral infections. Although establishing correlates of disease progression with hypermutation or expression levels as a surrogate of APOBEC antiviral functions has proven to be complex in HIV infection, *in vivo* defective proviral sequences clearly bear the hallmark of APOBEC editing. AID-APOBEC family members also participate in long-term adaptive immunity to viral infections. AID is critical for the generation of B cells that secrete high affinity Abs with various effector functions. A3G contributes in activating NK cell and CTL responses leading to the elimination of HIV-infected cells. Remarkably, the expression levels of AID-APOBEC family members are increased upon the sensing of infection and a tight regulation is required to avoid deleterious editing of host genomes.

Manipulating AID-APOBECs with drugs might constitute promising approaches to fight infections. Strategies have been proposed to increase A3 expression to favor encapsidation within viral particles thus overriding the antagonistic activity of Vif and controlling HIV-1 infection [reviewed in (Albin and Harris, 2010)]. The antiviral properties of Vif inhibitors are currently being evaluated (Ali et al., 2012). A3G expression can be manipulated either by limiting its proteasomal degradation (Ejima et al., 2011), or exacerbated to supraphysiologic levels. Treatment of HIV/HCV co-infected patients with IFN-α, for instance, increases mRNA expression of A3G/F and correlates with the degree of HIV hypermutation (Pillai et al., 2012). Upon vaccination, A3 expresssion can also be induced promoting adaptive immune responses (Wang et al., 2009; Sui et al., 2010). Taken together, these findings further highlight the complex and intriguing interactions of AID-APOBEC family members with the immune system.

## Conflict of Interest Statement

The authors declare that the research was conducted in the absence of any commercial or financial relationships that could be construed as a potential conflict of interest.
